# Supplementary material for: Snowy Dove: An open-sourcetoolkit for pre- processing of Chinese Gaofen series data
Source: PLoS One. 2024 Nov 13;19(11):e0313584. doi: 10.1371/journal.pone.0313584 (PMC11560028; doi:10.1371/journal.pone.0313584)
Supplement: S1 Appendix — (DOCX) [file pone.0313584.s001.docx]

# Appendix A Implementation of one-click pre-processing

Implementation in IDLDE

IDLDE can be opened by clicking the IDL icon under Windows or run the idlde command under *nix.

Following to the README file provided with Snowy Dove, there are 3 steps using Snowy Dove:

First, use the cd command (syntax: CD [, Directory]) to change the current working directory to the directory which stores the source codes of this software: i.e. type CD, ‘/home/dl/Snowy Dove/src’ and run this command to accomplish this step if the download path is /home/dl directory, in another words, the README file is in the /home/dl/Snowy Dove directory.

Second, run the commands starting with “.COMPILE” in the README file in order to compile the source code.

Third, use compiled procedure to pre-process massive data. The syntax is: sdMain, tgzdirIn [,DEM=demfn] [, REGION=shpfn] [, /CALI] [, /{QUAC | SCALE}] [, /TIFF] [, /NDVI] [, /PYRAMID] [, /VERBOSE] or sdMain, tgzdirIn [, d=demfn] [, r=shpfn] [, /c] [, /{q | s}] [, /t] [, /n] [, /p] [, /v].

Parameters in the syntax can be explained as follows:

tgzdirIn: directory stores *.tar.gz files

DEM (optional): filename of DEM used to orthorectify images

REGION (optional): filename of shapefile to subset images

CALI (optional): keyword to apply radiance calibration

QUAC (optional): keyword to apply QUAC

SCALE (optional): keyword to multiply the QUAC result with 0.0001

TIFF (optional): keyword to convert default ENVI format to TIFF format

NDVI (optional): keyword to get an extra NDVI result

PYRAMID (optional): keyword to build a pyramid file for output

VERBOSE (optional): keyword to print step in IDL console

To illustrate this, suppose you have some GF-1 WFV data (in directory /home/data) to pre-process, and the steps include orthorectification, radiometric correction, quick atmosphere correction, GeoTIFF export and NDVI export, the command may be sdMain, ‘/home/data’, /CALI, /QUAC, /TIFF, /NDVI

Implementation in the terminal

Snowy Dove can also fly in a terminal under the *nix operating system by running the executable file sdRunMain.sh using idl as interpreter. Usually, interpreter idl links to the default idl installation location /usr/local/exelis/idl/bin/idl. The syntax of executing sdRunMain.sh is similar to that in chapter 4.1 but in a more *nix flavor: idl ./sdRunMain.sh -args path [-r rpath] [-d dpath] -[cqscnpv]

# Appendix B Pseudocode

| Algorithm 1. Format Transfer | |
| --- | --- |
| Inputfile：ENVI image file (inputFile) | |
| Outputfile：GeoTIFF or BigGeoTIFF | |
| 1. | initialize image file header as IFH |
| 2. | write IFH to outputFile |
| 3. | initialize image file directory as IFD |
| 4. | if size of inputFile is bigger than 4G |
| 5. | while not encounter the end of inputFile |
| 6. | read one chunk of inputFile to chunkArray |
| 7. | write chunkArray to outputFile |
| 8. | else |
| 9. | read inputFile to entireArray |
| 10. | write entireArray to outputFile |
| 11. | write IFD to outputFile |
| 12. | get offset of IFD as ifdOffsets |
| 13. | write ifdOffsets to IFH |
| 14. | rewrite IFH to outputFile |

| Algorithm 2. Class linear scaling function |
| --- |
| Input：Dimensionless imagery file |
| Output：radiance image file(outputFile) |
| initialize gain and offet |
| for each channel in inputFile |
| read one channel as channelArray |
| write channelArray * gain + offset to outputFile |

| Algorithm 3. NDVI calculation |
| --- |
| Inpput：Multiband Image file(inputFile) |
| Output：normalized NDVI image file |
| for each line in inputFile |
| read one line as lineArray |
| compute ndvi from lineArray |
| write ndvi to outputFile |
