# Supplementary material for: Snowy Dove: An open-sourcetoolkit for pre- processing of Chinese Gaofen series data
Source: PLoS One. 2024 Nov 13;19(11):e0313584. doi: 10.1371/journal.pone.0313584 (PMC11560028; doi:10.1371/journal.pone.0313584)
Supplement: Attachment — Submitted filename: Response to reviewers.docx [file pone.0313584.s002.docx]

Reviewer #1:

Thank you for submitting your manuscript. After thorough review, I have identified several areas that require significant attention before considering your work for publication.

*Thank you very much for your careful review, we will definitely revise it carefully and strive to publish it as soon as possible.*

1. Clarity and Writing Quality:

The manuscript suffers from numerous grammatical errors, awkward phrasings, and unclear explanations throughout, making it challenging to follow the authors' reasoning and evaluate the methodology. A comprehensive revision of the language and structure is necessary to meet the standards of scientific publication. Please ensure clarity and coherence in your writing to enhance readability.

*We have made a complete revision of the language and structure of the manuscript, as well as the logical structure of the manuscript.*

2. Novelty and Significance:

The introduction lacks a comprehensive review of existing pre-processing methods for Chinese satellite data, making it unclear how Snowy Dove contributes to advancing the state-of-the-art. Please clearly articulate the specific limitations of current approaches and highlight the unique contributions of your work to establish its novelty and significance.

*In the introduction, we introduce a series of current preprocessing methods for domestic satellites, including the processing of each scene image by ENVI software, the extraction of grape growing areas by #C language, and the data processing of individual satellites by IDL language and so on. At present, there is no tool that can realize the batch processing of all the pretreatment processes of GF1/2/6, etc., which is the great significance of the white pigeon proposed in this study.*

3. Methodological Details:

The methodology section lacks important technical details on the algorithms implemented in Snowy Dove, particularly concerning the atmospheric correction approach. Please provide a more thorough explanation of your processing algorithms and workflow to enable proper evaluation and reproducibility.

*Thanks for the suggestion, we've added more technical details in the methods section, especially for atmospheric correction.*

4. Results and Analysis:

The results and analysis presented in the paper are insufficient to demonstrate the effectiveness and advantages of the Snowy Dove toolkit. Please ensure detailed captions and annotations for example images to facilitate interpretation of the improvements achieved by the software. Additionally, provide a more comprehensive and transparent analysis, including quantitative accuracy assessments of the geometric and radiometric corrections.

*Thanks to the suggestion, we have redrawn the picture of the article, and also supplemented it with a detailed introduction and quantitative analysis.*

5. Discussion Depth:

The discussion of the toolkit's characteristics and limitations lacks critical reflection and comparison with other existing software options. Please provide a meaningful analysis of how the capabilities of Snowy Dove compare to those of alternative tools and address potential limitations to enhance the depth of your discussion.

*In Tables 2 and 3, we have made a comparative analysis of the processing efficiency of different file sizes, and give a quantitative evaluation, which can highlight the important value of the Snowy Dove.*

6. Referencing:

The number and quality of references cited in the paper are insufficient. Please conduct a more thorough literature review and engage with relevant studies on satellite data pre-processing workflows, algorithm development, and accuracy assessment to contextualize your work within the broader field and support your methodological choices.

*Thanks for the comments, we have re-supplemented the exhaustive references, and we hope you are satisfied.*

In conclusion, while I recognize the potential value of Snowy Dove as an open-source toolkit for pre-processing Chinese satellite data, the manuscript requires substantial revisions to address the identified issues.

*Thank you for your recognition of Snowy Dove, according to the reviewers' comments, we have made comprehensive revisions and improvements to the manuscript.*

Reviewer #2:

i. The manuscript doesn't clarify what makes this toolkit unique. Are there existing pre-processing methods for Gaofen data? If so, how does this toolkit improve upon them?

*1.There are existing tools for the processing steps, like GUI tool ENVI. But the pro-processing steps are split into individual tools, users must use multiple tools to accomplish the pro-processing pipeline, and as each file size of a certain scene of Gaofen data is enormous, the processing time always cost hours in a GUI way. This toolkit will reduce the time between individual steps into nearly zero, and could processing as many data as user inputs.*

*2.The main improvement is that we optimize some algorithms from scratch, which is faster than the corresponding function in native ENVI/IDL. As for other processing steps, the toolkit is smart enough to choose a different way in order to save time, while ensuring the results are correct. For example, if the host OS is based on *nix, the de-compression step would use the `tar` toolkits instead of native IDL function.*

ii. To enhance the manuscript's impact, it would be beneficial to include a thorough comparison with existing pre-processing methods, showcasing the specific advantages and novel features of the proposed toolkit. This would strengthen the manuscript's originality and highlight its potential impact on the field

iii. The toolkit focus solely on pre-processing steps, potentially excluding image analysis or post-processing functionalities.

*Thanks for the suggestion. This toolkit do focus on pre-processing steps, which are less subjective than post-processing, and the post-processing steps differs across a range of application areas. So, we develop this toolkit first, and we will consider the later one.*

iv. To enhance the manuscript's clarity and usability, it is essential to provide detailed explanations of all preprocessing algorithms included in the toolkit. This would ensure that users would have have a comprehensive understanding of the algorithms and can implement them in their own work.

*Thanks for the suggestion. We provided some detailed explanations of all preprocessing algorithms included in the toolkit.*

Reviewer #3:

1. This paper's references and citations have several concerns. The authors only listed five sources.

*Thanks for the suggestions, we have update the references and add some latest citation.*

2. This paper discusses a number of concepts that either need a definition or a reference be included.

*Yes, we added both definition and reference for the concepts in the paper.*

3. Figure quality needs to be at least 300 dpi.

*Thanks for the suggestion. We have updated the figure in the manuscript.*

4. What are PAN and MSS? Which kind of statistical parameter is this? Why is it appropriate for this study?

*In the data part, we wrote “PAN and MSS represent panchromatic bands and multi-spectral band”， PAN and MSS are the two camera onboarded the GF1, GF2 and GF6. However the resolution of PAN and MSS of GF-2 is 1 m and 4 m, which is different from the 2 m and 8 m of GF-1 and GF-6. The data acquired by these two cameras was the main source of data for this study.*

5. As far as pre-processing goes, "APPENDIX A" only requires one click. For this implementation to be shown, the author should add the pseudocodes.

*Thanks for the suggestion, we added the pseudocodes as APPENDIX B, hope you are satisfied with the revised manuscript.*

6. What is the experiment on the testbed? This study requires a thorough correction, in particular the authors should add more citations and compare their findings both qualitatively and quantitatively with those of other comparable studies. Additionally, this study has to contain an experimental testbed in order to replicate this experiment by another researcher.

*We have tested this toolkit under Windows and Linux-based x86 operating systems, like Ubuntu and Fedora, etc. as for software environment, the basic one is the version of ENVI/IDL must be greater than 5.3/8.1, we tested different ENVI version, and if Python/GDAL installed. However, thanks for the cross-platform ability of ENVI/IDL and Python, this toolkit should run perfectly on macOS, too, we welcome Apple users pull their contributions. As we mentioned, ENVI/IDL, which is a commercial software package, plays an essential role in this toolkit, therefore, we could only provide source codes written in IDL with `.pro` filename suffix, which are also publicly available.*
