# Supplementary material for: Snowy Dove: An open-sourcetoolkit for pre- processing of Chinese Gaofen series data
Source: PLoS One. 2024 Nov 13;19(11):e0313584. doi: 10.1371/journal.pone.0313584 (PMC11560028; doi:10.1371/journal.pone.0313584)
Supplement: Attachment — Submitted filename: Response to reviewer.docx [file pone.0313584.s004.docx]

Reviewer #2: I recommend to integrate your previous responses into the revised manuscript (i.e. the novelty of your research and detail explanation of the algorithms and why you have chosen them.

Dear Reviewer #2,

Thank you for your valuable feedback and recommendation. I appreciate your suggestion to integrate my previous responses into the revised manuscript.

In response to your comment, I have revised the manuscript to include a more detailed explanation of the novelty of our research and the algorithms used in our toolkit. Specifically, I have highlighted how our toolkit integrates and optimizes Gaofen data preprocessing steps, reducing processing time and enabling batch processing for GF1/2/6 data, which is a significant advancement over existing tools like ENVI. Additionally, I have provided a more detailed explanation of the algorithms we have optimized from scratch and why we chose them, emphasizing their superiority in terms of speed and accuracy compared to the corresponding functions in native ENVI/IDL.

For raising the concern regarding the availability of all data found in our manuscript.

We understand your concern and want to assure you that we have taken steps to ensure that all relevant data and code are accessible to readers. As you mentioned, we have included the relevant code in the appendix of the manuscript. Additionally, we have provided a link to the data in the manuscript,(The GaoFen imagery used in this research is supported by the China Centre for Resources Satellite Data and Application (http://www.cresda.com, accessed on 5 July 2024).) which we believe provides easy access for interested readers to obtain the data for further analysis or verification.

We believe that providing the code and data in this manner adheres to best practices in research transparency and reproducibility. However, if there are specific data points or analyses that you believe are missing or not fully described, please let us know, and we will be happy to provide additional information or clarification as needed.

I believe these revisions will help to clarify the contributions of our research and strengthen the manuscript. Thank you again for your constructive feedback, and I look forward to your further comments on the revised manuscript.
